# NMR-Based Metabonomic Study Reveals Intervention Effects of Polydatin on Potassium Oxonate-Induced Hyperuricemia in Rats

**DOI:** 10.1155/2020/6943860

**Published:** 2020-07-05

**Authors:** Bin Han, Mengjuan Gong, Zhong Li, Yuqin Qiu, Zhongjie Zou

**Affiliations:** ^1^School of Traditional Chinese Medicine, Guangdong Pharmaceutical University, Guangzhou 510006, China; ^2^School of Pharmacy, Guangdong Pharmaceutical University, Guangzhou 510006, China

## Abstract

Previous studies have disclosed the antihyperuricemic effect of polydatin, a natural precursor of resveratrol; however, the mechanisms of action still remain elusive. The present study was undertaken to evaluate the therapeutic effects and the underlying mechanisms of polydatin on potassium oxonate-induced hyperuricemia in rats through metabonomic technology from a holistic view. Nuclear magnetic resonance (NMR) spectroscopy was applied to capture the metabolic changes in sera and urine collected from rats induced by hyperuricemia and polydatin treatment. With multivariate data analysis, significant metabolic perturbations were observed in hyperuricemic rats compared with the healthy controls. A total of eleven and six metabolites were identified as differential metabolites related to hyperuricemia in serum and urine of rats, respectively. The proposed pathways primarily included branched-chain amino acid (BCAA) metabolism, glycolysis, the tricarboxylic acid cycle, synthesis and degradation of ketone bodies, purine metabolism, and intestinal microflora metabolism. Additionally, some metabolites indicated the risk of renal injury induced by hyperuricemia. Polydatin significantly lowered the levels of serum uric acid, creatinine, and blood urea nitrogen and alleviated the abnormal metabolic status in hyperuricemic rats by partially restoring the balance of the perturbed metabolic pathways. Our findings shed light on the understanding of the pathophysiological process of hyperuricemia and provided a reference for revealing the metabolic mechanism produced by polydatin in the treatment of hyperuricemia.

## 1. Introduction

Hyperuricemia is characterized by a persistent increase of uric acid in circulating blood and is recognized as the key causal precursor in the development of gout [1]. Hyperuricemia is also believed to be an independent risk factor for metabolic syndrome and cardiovascular and renal diseases [2, 3]. Uric acid is produced from the oxidation of hypoxanthine and xanthine catalyzed by xanthine oxidase [4] and is the final product of purine metabolism in humans due to lack of uricase [5]. Thus, either the decreased excretion or the increased production of uric acid may lead to hyperuricemia [6]. With the change of lifestyle and dietary habits (excessive consumption of fructose, seafood, meat, and vegetables rich in purines), the incidence of hyperuricemia has increased rapidly, especially in adolescents and young adults [7].

Available urate-lowering agents can be grouped by their mechanism of action [8]: xanthine oxidase inhibitors (e.g., allopurinol), uricosuric agents (e.g., benzbromarone), and injectable uricases (e.g., pegloticase). However, concern on their tolerability and safety has been raised in recent times with a more attentive evaluation of pharmacovigilance studies [8]. For example, allopurinol has adverse reactions such as gastrointestinal symptoms and rash as well as allergic responses called allopurinol hypersensitivity syndrome (AHS) which can be life-threatening [9]. So, it is urgent to find more effective and safe alternative drugs to manage hyperuricemia.

As a natural precursor of resveratrol (resveratrol-3-O-*β*-mono-D-glucoside), polydatin is one of the main bioactive constituents isolated and identified from *Polygonum cuspidatum* Sieb. et Zucc., which is a commonly used traditional Chinese herbal medicine possessing analgesic and diuretic properties. A wide variety of biological activities of polydatin has been reported including cardiovascular effects, neuroprotection, anti-inflammatory and immunoregulatory effects, antioxidation, antitumor, and liver and lung protection [10]. Polydatin showed inhibitory activities on xanthine oxidase to repress the level of serum uric acid *in vivo* and *in vitro* [11]. In addition, polydatin could reduce serum urate levels by downregulating renal mURAT1 and mGLUT9 expression to inhibit urate reabsorption and downregulating mABCG2 with upregulation of mOAT1 and mOAT3 expression to increase urate secretion in the kidney of hyperuricemic mice [12, 13]. Furthermore, polydatin ameliorated renal injury by attenuating uric acid-induced inflammatory responses [11, 14]. However, further studies are needed to characterize underlying mechanisms by which polydatin acts on hyperuricemia.

Focusing on the changes of small molecular metabolites induced by environmental stimuli or perturbation, metabonomics can provide the global metabolic status of an entire organism [15] and has been widely used in the fields of disease diagnosis, toxicity assessment, evaluation of drug efficacy, and so on [16–18]. But little has been reported on the response of rat metabolic networks to the intake of polydatin from a holistic view. In the current study, a nuclear magnetic resonance- (NMR-) based approach was applied to explore therapeutic effects and underlying mechanisms of polydatin on potassium oxonate-induced hyperuricemia.

## 2. Materials and Methods

### 2.1. Chemicals and Reagents

Deuterium oxide (D_2_O, 99.9%), potassium oxonate, and allopurinol were obtained from Sigma-Aldrich (St. Louis, MO, USA). Polydatin (95%) was purchased from Shanghai Macklin Biochemical Co., Ltd. (Shanghai, China). All other chemicals used in this study were of analytical grade made in China.

### 2.2. Animals and Experimental Design

All of the experiments were approved by the Committee on the Ethics of Animal Experiments of Guangdong Pharmaceutical University, and all the procedures performed were strictly in accordance with the National Institutes of Health *Guide for the Care and Use of Laboratory Animals*. All efforts were made to reduce the number of animals used and to minimize any animal suffering.

Twenty-four adult male Sprague-Dawley (SD) rats weighing 200 ± 20 g were purchased at the Guangdong Provincial Medical Laboratory Animal Center (Foshan, Guangdong, China). Rats were housed in a SPF grade experimental animal facility with food and water freely available. After one week of acclimatization, rats were randomly assigned to four groups (*n* = 6 rats/group): normal control group (C), hyperuricemia model group (M), polydatin-treated group (P), and allopurinol-treated group (A). Rats in the M, P, and A groups were treated with potassium oxonate (250 mg/kg) once a day for a total of 7 days while those in the C group received an equal volume of vehicle (0.5% CMC-Na). Polydatin (50 mg/kg) or allopurinol (5 mg/kg) was administrated to rats daily by gastric instillation 1 h after the potassium oxonate was given. The doses of drugs were set based on previous reports [14]. After dosing on day 6, rats were housed in metabolism cages individually and 12 h urine samples from each rat (about 4-9 ml) were collected into ice-cooled vessels containing 0.5 ml of 2% sodium azide as a bacteriostatic agent followed by centrifugation at 5000 rpm at 4°C for 10 min. Three hours after the last dosing on day 7, blood was collected from the retroorbital venous plexus after rats were anesthetized by diethyl ether inhalation, and serum was obtained by centrifuging at 4000 rpm for 10 min after blood clotting for 1 h at 4°C. All of the samples were stored at -80°C until biochemical determinations and metabonomic analysis.

### 2.3. Serum Biochemical Assay

The levels of serum uric acid (SUA), creatinine (Scr), and blood urea nitrogen (BUN) were measured based on an enzymatic-colorimetric method using commercially available kits (Nanjing Jiancheng Bioeng. Inst., Nanjing, China) according to the manufacturer's instructions. Data were expressed as the mean ± SD and analyzed by one-way analysis of variance (ANOVA) followed by Tukey's post hoc test for multiple comparisons using SPSS 20.0 (SPSS Inc., Chicago, IL, USA). Values of *P* less than 0.05 were considered to be statistically significant.

### 2.4. Sample Preparation for Metabonomic Analysis

Samples were prepared according to our previously published procedures [19]. After samples were thawed, 50 *μ*l of phosphate-buffered solution (0.2 M Na_2_HPO_4_ and 0.2 M NaH_2_PO_4_, pH 7.4) and 50 *μ*l of D_2_O were added to 400 *μ*l of serum. Then, the serum samples were centrifuged at 4000 rpm at 4°C for 10 min, and the supernatants were transferred into 5 mm NMR tubes. For urine samples, 400 *μ*l of urine was thoroughly mixed with 200 *μ*l of phosphate buffer (0.2 M Na_2_HPO_4_-0.2 M NaH_2_PO_4_, pH 7.4). After standing at 4°C for 20 min, urine samples were centrifuged (4000 rpm, 10 min, 4°C) and supernatants were collected for further analysis. 500 *μ*l of the supernatants from each urine sample was transferred into 5 mm NMR tubes with an addition of 50 *μ*l of D_2_O containing 0.05% (*w*/*v*) of sodium 3-trimethylsilyl [2,2,3,3-d4] propionate (TSP-*d_4_*) as a chemical shift reference (*δ*0).

### 2.5. NMR Data Acquisition

^1^H-NMR spectra of serum and urine samples were recorded at 298 K on a Bruker AVANCE III 500 MHz spectrometer (Bruker BioSpin, Rheinstetten, Germany) operating at 500.13 MHz ^1^H frequency. 128 free induction decays (FIDs) were collected into 64K data points over a 10000 Hz spectral width for each sample. For serum samples, the water-suppressed Carr-Purcell-Meibom-Gill (CPMG) spin-echo pulse sequence (RD‐90‐(*τ*‐180*τ*)_*n*_‐ACQ) with a total spin-echo delay (2*nτ*) of 100 ms was used to attenuate broad signals from proteins and lipoproteins. A standard 1D nuclear Overhauser enhancement spectroscopy- (NOESY-) presaturation pulse sequence was used to acquire ^1^H NMR spectra of urine. A relaxation delay of 3.0 and an acquisition time of 3.28 s were set [19].

### 2.6. NMR Data Processing

All acquired NMR spectra were processed with a line-broadening factor of 0.3 Hz prior to Fourier transformation and subsequent manual phase and baseline correction using MestreNova 6.1 software (Mestrelab Research S.L., Santiago de Compostela, Spain). All resulting spectra were divided, and the signal integral was computed in 0.01 interval across the region *δ*0.50-9.50 after the ^1^H NMR spectra were referenced to methyl resonance of lactate (*δ*1.33) for serum and TSP (*δ*0.0) for urine. The regions of *δ*4.68-5.22 and *δ*4.50-5.96 from serum and urine spectra, respectively, were excluded to eliminate the effects of water. Finally, before pattern recognition, all remaining integral regions of the spectra were normalized to the integrated area of the spectra and then multiplied by 10000.

After Pareto scaling and mean centering, the acquired integral data were used for multivariate data analysis such as partial least-squares discriminant analysis (PLS-DA) with SIMCA-P 12.0 software (Umetrics, Umea, Sweden) to clarify the difference between groups. The model parameters including *R*^2^ (goodness of fit) and *Q*^2^ (goodness of prediction) calculated from the PLS-DA models were used to assess the quality of these models. Metabolites with both values of variable importance in the projection (VIP) ≥ 1.0 obtained from PLS-DA models and values of *P* < 0.05 from an independent sample *t*-test or Mann-Whitney *U* test were considered as differential metabolites.

### 2.7. Pathway Analysis

Metabolic pathway analysis of differential metabolites associated with hyperuricemia in rats was performed with MetaboAnalyst (http://www.metaboanalyst.ca/). Pathways with the impact value > 0.1 were selected as significantly perturbed metabolic pathways in rats with hyperuricemia [20].

## 3. Results

### 3.1. Effects of Polydatin on the Levels of SUA, Scr, and BUN in Hyperuricemic Rats

**Figure 1:**
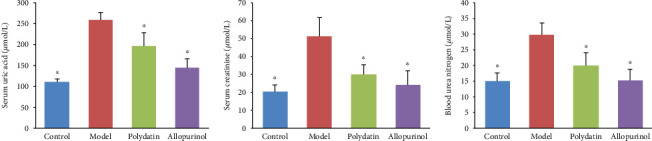
Polydatin decreased the levels of serum uric acid (SUA), creatinine (Scr), and blood urea nitrogen (BUN) in rats with hyperuricemia. Values are given as the mean ± SD (*n* = 6), ^∗^*P* < 0.05 compared with the hyperuricemia model group.

Elevated SUA is the major characteristic of hyperuricemia. BUN and Scr are nitrogenous end products of protein metabolism, and their levels are valuable markers of renal function. As shown in Figure 1, orally administrated potassium oxonate significantly increased the levels of SUA, Scr, and BUN compared with normal rats. Both polydatin at 50 mg/kg and allopurinol (5 mg/kg) as a positive control significantly reduced the levels of SUA, Scr, and BUN in hyperuricemic rats, consistent with previous studies [14]. These results demonstrated that polydatin exerts beneficial effects on hyperuricemia in rats.

### 3.2. Metabolic Perturbations and Differential Metabolites Associated with Hyperuricemia in Rats

**Figure 2:**
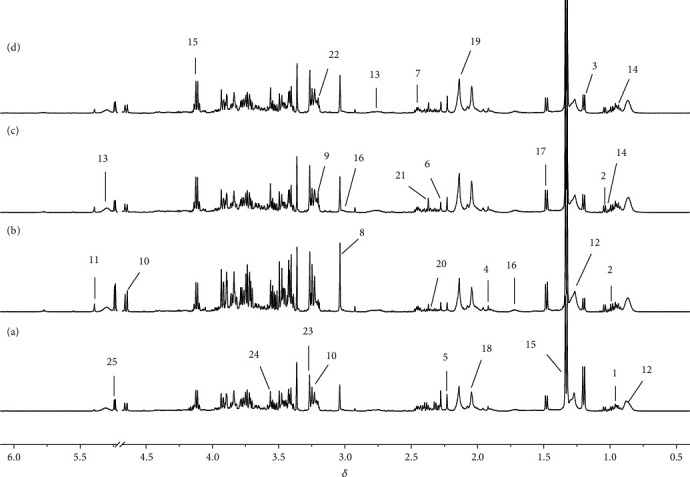
Typical ^1^H NMR spectra of serum samples of rats from control (a), model (b), polydatin (c), and allopurinol (d) groups. Keys: 1: leucine; 2: valine; 3: 3-hydroxybutyrate; 4: acetate; 5: acetone; 6: acetoacetate; 7: glutamine; 8: creatine/phosphocreatine; 9: phosphocholine; 10: *β*-glucose; 11: allantoin; 12: VLDL/LDL; 13: unsaturated lipids; 14: isoleucine; 15: lactate; 16: lysine; 17: alanine; 18: N-acetyl glycoproteins; 19: O-acetyl-glycoproteins; 20: glutamate; 21: pyruvate; 22: choline; 23: betaine/trimethylamine N-oxide; 24: glycine; 25: *α*-glucose.

**Figure 3:**
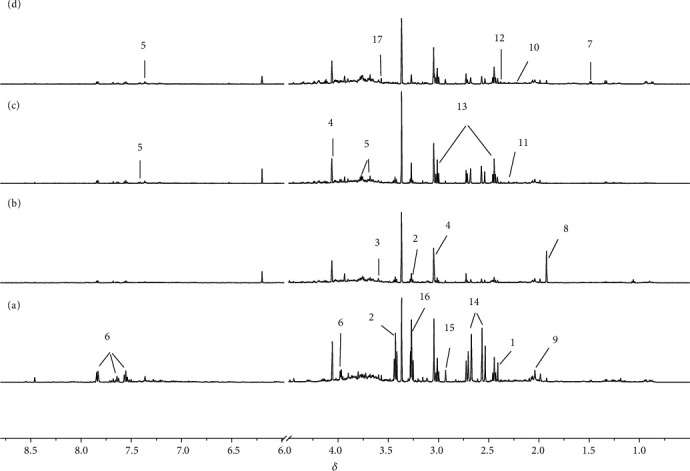
Typical ^1^H NMR spectra of urine samples of rats from control (a), model (b), polydatin (c), and allopurinol (d) groups. Keys: 1: succinate; 2: taurine; 3: sarcosine; 4: creatinine; 5: phenylacetylglycine; 6: hippurate; 7: alanine; 8: acetate; 9: N-acetyl glycoproteins; 10: acetone; 11: acetoacetate; 12: pyruvate; 13: 2-oxoglutarate; 14: citrate; 15: dimethylglycine; 16: betaine/trimethylamine N-oxide; 17: glycine.

**Figure 4:**
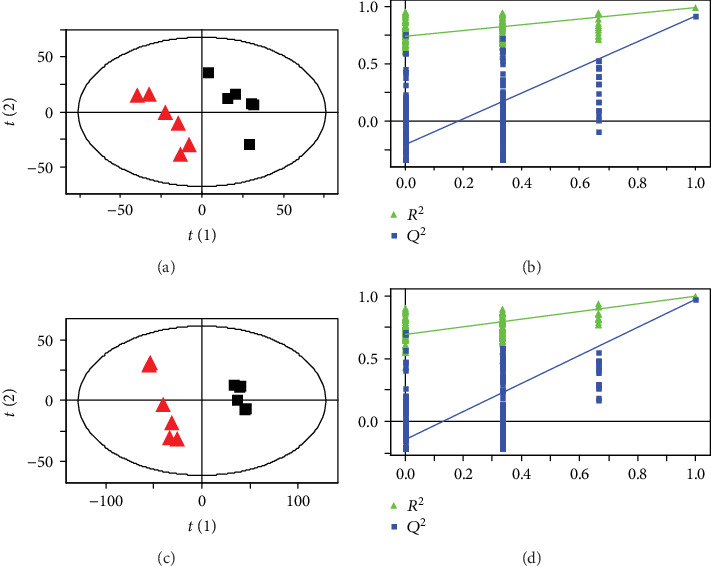
PLS-DA score plots (left) and the corresponding validation plots (right) with 200 times permutation tests obtained from ^1^H NMR spectra of rat serum (a, b) and urine (c, d). ■: control group; ▲: hyperuricemia model group.

The representative ^1^H NMR spectra obtained from serum and urine samples are shown in Figures 2 and 3, respectively. The resonances of major endogenous metabolites were identified by referencing to the Human Metabolome Database (HMDB) and relevant literature [21, 22] and with the aid of the Chenomx NMR suite (Chenomx Inc., Edmonton, AB, Canada). As shown in the PLS-DA score plots of serum and urine samples (Figures 4(a) and 4(c)) based on the normalized NMR spectral data, a separation between the model group and the control group was clearly seen, indicating that the hyperuricemia model was successful and had completely different metabolic profiles compared with the healthy controls. The parameters of the PLS-DA models were as follows: *R*^2^*Y* = 0.97 and *Q*^2^ = 0.91 for serum; *R*^2^*Y* = 0.99 and *Q*^2^ = 0.98 for urine. Generally, it was supposed to be an excellent model when values of *R*^2^*Y* and *Q*^2^ were greater than 0.8 [23]. In addition, 200-iteration permutation tests were also performed to assess the robustness of these PLS-DA models. The validation plots showed that the original PLS-DA models were not random and overfitted as both permutated *Q*^2^ and *R*^2^ values were significantly lower than the corresponding original values along with the *Y*-intercepts of the regression lines of the *Q*^2^-points below zero (Figures 4(b) and 4(d)).

Discriminatory metabolites listed in Table 1 were determined by referring to VIP values from the OPLS-DA models (VIP ≥ 1) and *P* values from univariate statistical analysis (*P* < 0.05). A total of 11 and 6 metabolites were, respectively, identified as differential metabolites related to hyperuricemia in serum and urine. Compared with the control group, the metabolic perturbations occurring in serum of the hyperuricemic rats were mainly characterized by decreased levels of leucine, valine, 3-hydroxybutyrate, acetate, acetone, acetoacetate, glutamine, and phosphocholine, along with increased levels of creatine/phosphocreatine, *β*-glucose, and allantoin. Hyperuricemic rats also showed greater urinary levels of sarcosine and creatinine and lower levels of succinate, taurine, phenylacetylglycine, and hippurate in comparison to normal rats.

### 3.3. Metabolic Changes under the Treatment of Polydatin and Allopurinol

**Figure 5:**
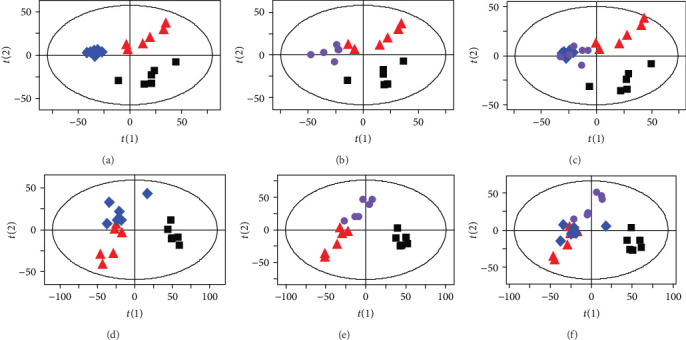
Metabolic profiles of rat serum (a–c) and urine (d–f) in the control (∎), model (▲), polydatin (♦), and allopurinol (●) groups.

**Figure 6:**
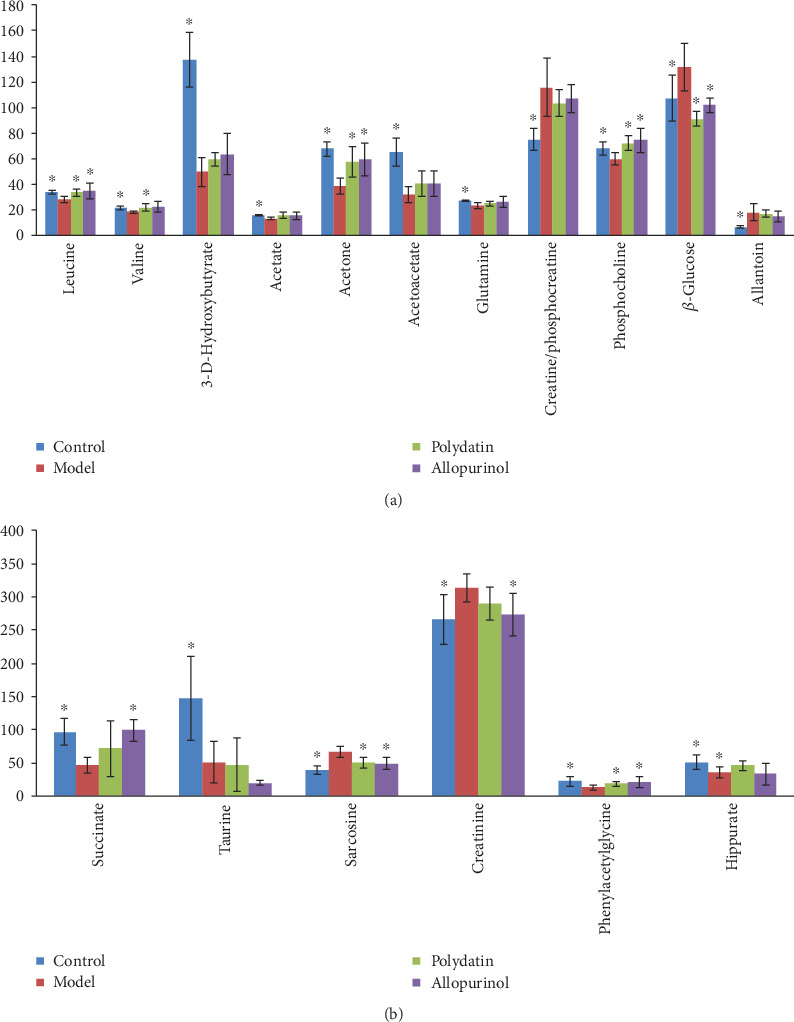
Relative levels of differential metabolites related to hyperuricemia in rat serum (a) and urine (b). Error bars indicate the mean ± SD (*n* = 6). ∗ represents *P* < 0.05 compared with the hyperuricemia model group.

Figure 5 showed distinct metabolic profiles among different groups and also a tendency to return to the normal group in polydatin-treated and allopurinol-treated groups. Furthermore, five and three differential metabolites associated with hyperuricemia in serum and urine samples of rats were significantly reversed by polydatin, respectively. Administration of allopurinol attenuated alteration of a total of eight differential metabolites in serum and urine samples (Figure 6 and Table 1). These findings suggested that the metabolic perturbations induced by hyperuricemia could be normalized by polydatin and allopurinol treatment. However, there were differences in the potential biomarkers reversed by puerarin and allopurinol, which might be attributable to the distinct mechanisms of action.

## 4. Discussion

Polydatin exhibits an extensive range of biological activities, and antihyperuricemic effects of polydatin have recently been investigated [10, 14]. To the best of our knowledge, our study was the first to explore the beneficial effects of polydatin on hyperuricemic rats using NMR-based metabolomics from a holistic view. Data and results of the study provided an in-depth and comprehensive understanding of hyperuricemia and the metabolic effects of polydatin.

**Figure 7:**
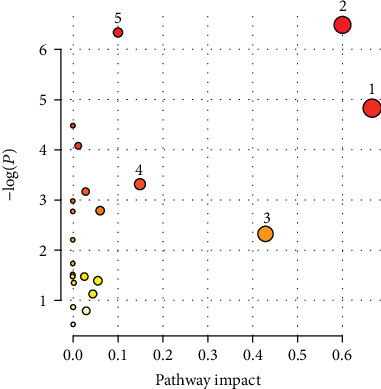
Summary of pathway analysis. (1) Valine, leucine, and isoleucine biosynthesis; (2) synthesis and degradation of ketone bodies; (3) taurine and hypotaurine metabolism; (4) alanine, aspartate, and glutamate metabolism; (5) butanoate metabolism.

In clinical practice, diagnosis of hyperuricemia is based on significantly elevated SUA levels alone, which hinders its early detection and prevention. Therefore, identification of more biomarkers potentially associated with hyperuricemia is crucial. In our study, eleven and six metabolites in serum and urine samples of rats, respectively, are identified as differential metabolites related to hyperuricemia, and five significantly disturbed metabolic pathways including valine, leucine, and isoleucine biosynthesis; synthesis and degradation of ketone bodies; taurine and hypotaurine metabolism; alanine, aspartate, and glutamate metabolism; and butanoate metabolism are revealed (Figure 7).

Previous studies have consistently demonstrated the dysfunctional energy metabolism in patients with hyperuricemia as well as in animal models [22, 24–26]. Important tricarboxylic acid (TCA) cycle intermediate metabolites such as succinate, citrate, and fumarate were downregulated in urine of gout patients and hyperuricemic mouse [27, 28]. Hyperuricemic rats in our study exhibited an elevated level of *β*-glucose in serum together with a decreased level of succinate in urine as compared with normal rats. These data suggest changes in carbohydrate metabolism. A large number of studies have shown that serum uric acid is positively correlated with elevated blood glucose due to insulin resistance [29, 30], and the occurrence of hyperuricemia is often associated with type 2 diabetes mellitus [3]. Succinate is a key intermediate of the TCA cycle in mitochondria which involves not only the glucose aerobic oxidation but also the major pathways for fat and amino acid metabolisms. Therefore, our results demonstrated the inhibition of glycolysis and TCA oxidation as the source of energy supply induced by hyperuricemia in rats. Meanwhile, NMR spectroscopy measurements of serum samples highlighted a decrease in *β*-hydroxybutyrate, acetone, and acetoacetate in hyperuricemic rats. In healthy individuals, the body uses mostly carbohydrate metabolism to fuel its cells. If sufficient carbohydrates are not available, the body begins to metabolize fats into ketone bodies (*β*-hydroxybutyrate and acetoacetate) to provide the necessary fuel. Lipidomics studies revealed that saturated fatty acid LPCs were upregulated and unsaturated fatty acid LPCs were downregulated in potassium oxonate-induced hyperuricemic rats [31], while increased levels of all PCs and unsaturated fatty acid LPCs together with decreased levels of saturated fatty acid LPCs were detected in rats with hyperuricemia induced by fructose [32]. These contrary conclusions might be due to different model-making methods. In the present study, the decrease in ketone body levels implied that, on the one hand, ketone bodies provided an alternative energy source to make up for the suppression of glycolysis and TCA cycle activity and, on the other hand, the abnormal lipid metabolism mentioned above led to the dysfunction of ketone body synthesis.

Aberrant amino acid metabolism has been previously reported in the plasma of patients with hyperuricemia [33]. Amino acids play important roles in multiple physiological and pathophysiological processes including the biosynthesis of uric acid. Accumulating evidences show the linkage between amino acids and metabolic syndrome, insulin resistance, hypertriglyceridemia, and type 2 diabetes [34, 35]. Luo et al. showed that isoleucine, lysine, and alanine could be the potential markers to distinguish patients with gout from hyperuricemia [33]. The serum levels of branched-chain amino acids (BCAAs) leucine and valine were decreased in the model group. Rats with hyperuricemia induced by fructose showed decreased levels of isoleucine and valine in plasma [36]. However, contrary to our results, elevated levels of leucine and valine in serum were observed in gout patients [37]. The discrepant results may be due to differences in metabolism between humans and animals. Glutamine is an important material for purine biosynthesis through the de novo synthesis pathway [9], and its serum level would affect the production of uric acid [38]. A significant reduction in the serum glutamine level in hyperuricemic rats in the present study revealed the acceleration of purine anabolism. Uric acid could induce the generation of reactive oxygen species (ROS) leading to oxidative stress [39]. Wang et al. reported the decreased taurine level in urine and plasma of hyperuricemic rats [22]. Taurine is an antioxidant and an organic osmolyte capable of protecting and stabilizing cells [20]. The decrease in urinary taurine might reflect increased taurine consumption to defend against oxidative damage induced by elevated uric acid. Nephropathy is a common complication of hyperuricemia. Creatinine, a breakdown product of creatine, is produced at a constant rate, and raised urinary creatinine is observed only in the case of damaged renal function [22, 26]. As markers of renal function, BUN and Scr levels were elevated in model rats in this study. Consistent with previous studies [22], increased excretion of creatinine in urine was observed in hyperuricemic rats, indicating renal injury caused by hyperuricemia. In addition, imbalanced amino acid metabolism has been reported to be associated with chronic kidney disease [40]. Decreased glutamine was observed in the present study and rats with hyperuricemia-induced nephropathy [41]. Glutamine plays an essential role in the regulation of acid-base homeostasis with glutamine being the key donor of NH_3_ in the kidney. Under the hyperuricemia condition, it is very likely that the kidney takes up more glutamine in order to compensate for the excess acid. Enhancement of renal extraction of glutamine took place during acidosis [42]. The decreased level of glutamine might indicate abnormal kidney function.

Uric acid is decomposed into soluble allantoin in most mammals except for higher primates because of the lack of uricase [5]. Although potassium oxonate, the inhibitor of uricase, was used to establish the hyperuricemic model in our study, the uricase could still catalyze the oxidation of uric acid to allantoin to some degree, and the level of allantoin could in part represent the amount of uric acid. Allantoin was found to be elevated in urine and feces of hyperuricemic rats [22, 26, 41]. The model rats showed a greater serum level of allantoin than the controls. Furthermore, allantoin was believed to be a biomarker for oxidative stress [43], and the level of allantoin in serum was elevated in the model group possibly due to oxidative damage induced by uric acid.

It is well known that the gut microbiome could be partially responsible for converting xanthine into uric acid and excreting 1/3 of the uric acid into the intestinal tract [44]. The gut microbiota and its metabolites play significant roles in the pathogenesis of hyperuricemia-related diseases, such as gout [45]. Compared to the control rats, hippurate and phenylacetylglycine, uniquely produced by bacterial metabolism in the intestinal tract [46], were significantly decreased in urine in hyperuricemia model rats, depicting the possible implication of gut microbiota disturbance in the pathogenesis of hyperuricemia. Alterations of gut microbiota revealed the possible molecular mechanism of nephropathy induced by hyperuricemia [41] and were associated with the treatment of hyperuricemia in rats [47].

Previous studies showed that inhibition of xanthine oxidase activity, promotion of renal uric acid excretion, and suppression of the inflammatory cascade were implicated in the therapeutic effect of polydatin on hyperuricemia in rats. In our study, administration of polydatin reversed a total of eight differential metabolites associated with hyperuricemia in model rats. These results implied that polydatin exerted antihyperuricemia activities by partially restoring the balance of the perturbed metabolic pathways, including branched-chain amino acid (BCAA) metabolism, glycolysis, the tricarboxylic acid cycle, synthesis and degradation of ketone bodies, purine metabolism, and intestinal microflora metabolism.

## 5. Conclusion

In this work, a metabolomic approach based on NMR is developed to profile serum and urine metabolic changes related to potassium oxonate-induced hyperuricemia in rats and investigate the antihyperuricemia effect of polydatin. A total of eleven and six metabolites are identified as differential metabolites related to hyperuricemia in serum and urine samples of rats, respectively. Polydatin lowers the serum level of uric acid through intervening on these metabolic dysfunctions. These findings shed light on the understanding of the pathophysiological process of hyperuricemia and provided a reference for revealing the metabolic mechanism produced by polydatin in the treatment of hyperuricemia.

## Figures and Tables

**Table 1:** Identification results of differential metabolites associated with hyperuricemia in rats.

Biological matrices	Metabolites	Chemical shift (ppm)^a^	VIP^b^	M *vs.* C^c^	P *vs.* M^c^	A *vs.* M^c^
Serum	Leucine	0.96(t)	1.9	↓^∗^	↑^∗^	↑^∗^
	Valine	0.99(d), 1.04(d)	1.4	↓^∗^	↑^∗^	–
	*β*-Hydroxybutyrate	1.20(d)	7.6	↓^∗^	–	–
	Acetate	1.92(s)	1.3	↓^∗^	–	–
	Acetone	2.23(s)	2.3	↓^∗^	↑^∗^	↑^∗^
	Acetoacetate	2.28(s)	4.6	↓^∗^	–	–
	Glutamine	2.45(m)	1.4	↓^∗^	–	–
	Creatine/phosphocreatine	3.04(s)	4.8	↑^∗^	–	–
	Phosphocholine	3.21(s)	2.0	↓^∗^	↑^∗^	↑^∗^
	*β*-Glucose	3.25(dd), 4.65(d)	3.4	↑^∗^	↓^∗^	↓^∗^
	Allantoin	5.40(s)	2.6	↑^∗^	–	–

Urine	Succinate	2.41(s)	2.6	↓^∗^	–	↑^∗^
	Taurine	3.26(t), 3.42(t)	4.0	↓^∗^	–	–
	Sarcosine	3.60(s)	2.3	↑^∗^	↓^∗^	↓^∗^
	Creatinine	4.06(s)	4.8	↑^∗^	–	↓^∗^
	Phenylacetylglycine	3.68(s), 3.76(d), 7.35(m), 7.43(t)	1.1	↓^∗^	↑^∗^	↑^∗^
	Hippurate	3.97(d), 7.56(t), 7.64(t), 7.84(d)	1.4	↓^∗^	↑^∗^	–

^a^Letters in parentheses indicate the peak multiplicities: s: singlet; d: doublet; t: triplet; q: quartet, m: multiplet. ^b^VIP was obtained from PLS-DA models (Figures 4(a) and 4(c)). ^c^∗ represents *P* < 0.05, whereas – denotes no statistically significant difference. ↑ indicates a relative increase in the signal, while ↓ indicates a relative decrease in the signal. C: normal control group; M: hyperuricemia model group; P: polydatin-treated group; A: allopurinol-treated group.

## Data Availability

The data used to support the findings in this paper are available from the corresponding author upon request.
